# Critical Motor Number for Fractional Steps of Cytoskeletal Filaments in Gliding Assays

**DOI:** 10.1371/journal.pone.0043219

**Published:** 2012-08-21

**Authors:** Xin Li, Reinhard Lipowsky, Jan Kierfeld

**Affiliations:** 1 Max Planck Institute of Colloids and Interfaces, Science Park Golm, Potsdam, Germany; 2 Physics Department, TU Dortmund University, Dortmund, Germany; German Cancer Research Center, Germany

## Abstract

In gliding assays, filaments are pulled by molecular motors that are immobilized on a solid surface. By varying the motor density on the surface, one can control the number 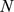 of motors that pull simultaneously on a single filament. Here, such gliding assays are studied theoretically using Brownian (or Langevin) dynamics simulations and taking the local force balance between motors and filaments as well as the force-dependent velocity of the motors into account. We focus on the filament stepping dynamics and investigate how single motor properties such as stalk elasticity and step size determine the presence or absence of fractional steps of the filaments. We show that each gliding assay can be characterized by a critical motor number, 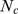. Because of thermal fluctuations, fractional filament steps are only detectable as long as 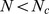. The corresponding fractional filament step size is 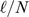 where 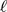 is the step size of a single motor. We first apply our computational approach to microtubules pulled by kinesin-1 motors. For elastic motor stalks that behave as linear springs with a zero rest length, the critical motor number is found to be 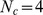, and the corresponding distributions of the filament step sizes are in good agreement with the available experimental data. In general, the critical motor number 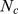 depends on the elastic stalk properties and is reduced to 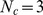 for linear springs with a nonzero rest length. Furthermore, 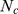 is shown to depend quadratically on the motor step size 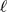. Therefore, gliding assays consisting of actin filaments and myosin-V are predicted to exhibit fractional filament steps up to motor number 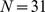. Finally, we show that fractional filament steps are also detectable for a fixed *average* motor number 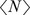 as determined by the surface density (or coverage) of the motors on the substrate surface.

## Introduction

Molecular motors are enzymes which convert chemical energy into mechanical work. Motor proteins such as kinesin, dynein or myosin are unidirectional stepping motors, which are involved in force generation and active intracellular transport. Kinesin-1 and myosin-V are processive motors moving on microtubule (MT) or actin filaments, respectively, for example for intracellular cargo transport. Such cargo transport often involves groups of cooperating motor proteins. Whereas the stepping mechanism of single motor proteins is well-studied experimentally and theoretically, much less is known about the resulting cargo step sizes in collective transport. In a recent experiment by Leduc *et al.* cargo step sizes have been studied in gliding assays with kinesin-1 motor proteins and MTs [1].

In gliding assays, the tails of molecular motors are immobilized on a planar substrate while their motor heads attach to filaments and pull them over the substrate [2], [3]. In the gliding assays of Leduc *et al.*, labeled MTs were observed to perform stepwise motion as a result of the transport by stepping kinesin-1 motors with step size 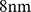. Monitoring the filament rotation it was possible to discriminate between transport by (i) one motor (ii) two motors and (iii) more than two motors and analyze MT trajectories separately for these cases. In this analysis an 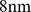 MT step size was found for MTs transported by a single motor, whereas half steps of 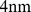 were found when MTs were transported cooperatively by two kinesin-1 motors, whereas smaller fractional step sizes such as 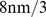 for transport by more than two kinesin-1 motors have not been observed. As explained further below, additional noise in the experiments reduces the critical motor number below which fractional steps can be observed to 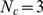. On the one hand, the observation of fractional filament steps provides evidence that kinesin-1 stepping in cooperative transport is not synchronized. On the other hand, it remains to be understood which system properties determine the presence or absence of higher-order fractional steps and whether fractional filament stepping can be expected for gliding assays with other processive motors such as myosin-V.

In order to address these latter issues, we describe the gliding assays by microscopic Brownian (or Langevin) dynamics [4], [5]. In this latter dynamics, we numerically solve the equations of motion for the translation and rotation of a rigid filament under the influence of the forces arising from the attached molecular motors as well as from thermal and frictional forces [6]–[8]. We focus on gliding assays for the processive motors kinesin-1 (henceforth called “kinesin”) and myosin-V with long run lengths. Kinesins walk along MTs towards their plus end with a step size of 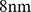 [9], whereas myosin-V walks along actin filaments with a much larger step size of 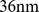 [10], [11]. Our theoretical description contains several microscopic properties of motor proteins: the step size, a force-dependent stepping frequency or velocity, motor stalk length and stalk stiffness, rates for force-free attachment and detachment of motor heads to and from the filament, as well as a detachment force for force-induced motor detachment. For gliding assays of microtubules and kinesin, we use the kinesin motor parameters as reported in Ref. [1]. Because analogous experimental data on actin/myosin-V gliding assays are not available, we use literature values from different sources for myosin-V. In particular, the length and elasticity of the motor stalk are taken from literature values for mouse myosin-V [12], [13]. We use a coarse-grained description in the sense that we do not resolve the two distinct motor domains of the double-headed motors.

Motors pulling on the same filament take steps in an unsynchronized manner but these steps generate mutual load forces and, thus, correlations between the motors. Indeed, each motor step gives rise to an instantaneous load force within the polymeric motor stalk, which is described by the force-extension relation of the stalk. This load force is transmitted onto the filament and then affects, via the resulting filament motion, all attached motor heads and their stepping frequency.

In addition to the microscopic Brownian dynamics, we also study the fractional steps of the filaments using a simplified description in terms of a force equilibrium model, in which the elastic forces of the motor stalks are mechanically balanced after each motor step. The force equilibrium model shows that motor step size, motor stalk length, and motor stalk elasticity are the essential motor parameters determining fractional filament stepping.

Using the microscopic simulation model we investigate the resulting stepwise motion of the transported filaments. In particular, we study how the number 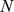 of attached motors and the elastic properties of the motor stalks, which are responsible for the force transduction from motor heads onto the filament, affect the stepwise motion of filaments. There are only a few experimental results on the elasticity of motor stalks for kinesin [14]–[16] and myosin-V [12]. Therefore, we will study the influence of the elastic properties of the motor stalk on the filament stepping behavior. The motor stalk consists of polypeptide chains, and we will compare four generic models from polymer physics for the elastic properties of the motor stalk [6], [17]: (I) a simple linear Hookian spring with zero rest length, (II) a linear spring with non-zero rest length, (III) a nonlinear spring for a freely jointed chain without bending energy, where chain segments are connected fully flexible, and (IV) a nonlinear spring for a worm-like chain with bending rigidity.

For kinesin gliding assays our microscopic simulation model achieves *quantitative* agreement with the experimentally observed MT stepping behavior in Ref. [1] even for the shapes of MT step size distributions. This agreement is remarkable because we use much higher motor velocities as appropriate for physiological ATP concentrations, whereas the experiments were performed for very low ATP concentration and, thus, rather low motor velocities in order to reduce additional noise from the MT position measurements by quantum dot position tracking. Our simulation results therefore show that the experimentally observed stepping behavior applies to a larger range of parameters. We find that fractional half-steps of 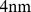 for transport by 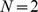 kinesin motors occur for all four variants (I) – (IV) of the elastic motor stalks, a property that we can also understand in the framework of the force equilibrium model. On the other hand, smaller fractional step sizes of 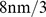 for MTs transported by 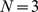 motors occur only for the elastic springs (I), (III), and (IV), which all have a zero rest length. For the linear spring (II) with non-zero rest length, we do not find smaller fractional step sizes than 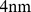 because of much broader distributions of step sizes. This broadening can also be understood in the framework of the force equilibrium model.

We then consider gliding assays in general, i.e., built up from an arbitrary pair of cytoskeletal filaments and motors. We show that each such pair can be characterized by a critical motor number, 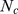. Because of thermal fluctuations, fractional filament steps are only detectable as long as 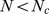. For motor stalks that act as linear springs with spring constant 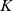, we derive an explicit expression for the critical motor number 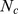, which is found to be proportional to the spring constant 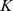 and to the *squared* step size of a single motor. For kinesin motors with a step size of 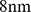, we find 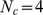 and fractional steps become undetectable for 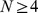 in agreement with our simulation results. We also study gliding assays of actin filaments pulled by myosin-V motors, which have the larger step size 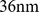. For this latter system, our simulations reveal fractional steps up to the much higher motor number 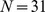, in agreement with our explicit expression for the threshold number.

So far, we have implicitly assumed that the overall filament trajectories can be decomposed into distinct segments, each of which is characterized by a fixed motor number 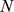. Such a decomposition is always possible in simulations and has also been achieved experimentally in Ref. [1] up to 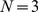. However, it is hardly possible to experimentally distinguish segments with 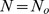 from those with 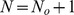 for large values of 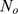. In contrast, the *average* number 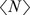 can be directly controlled experimentally via the surface density (or coverage) of the motors on the substrate surface. Thus, at the end, we also determine the step size distributions of filaments for fixed average number 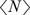 and find, for a wide range of 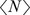-values, that these distributions exhibit fractional filament steps as well.

## Methods

Our microscopic simulation model is based on a gliding assay model which has been introduced in Refs. [4], [5]. Here we use this model to study the motor-driven motion of a single rigid filament in the two-dimensional substrate plane. We use the same model with different parameters both for kinesin/MT and myosin-V/F-actin gliding assays. We include stochastic discrete motor stepping into this model, which is essential for filament stepping dynamics.

The simulation model contains three types of degrees of freedom: (i) the filament configuration as described by its center of mass and orientation in the two-dimensional substrate plane; (ii) motor heads, which can attach and move on the filament and are described by their position; (iii) motor stalks, which are stretched by the motion of motor heads and transmit their stretching forces both onto the filament and the motor head. To understand the origin of fractional filament steps it is crucial that the simulation model explicitly includes motor stalks, which act as force transducers and are modeled as polymeric springs. These springs are characterized by a force-extension relation, which specifies the stretching force for a given equilibrium distance between the substrate-anchored motor tail and the motor head.

In order to simulate the filament motion we use Brownian (or Langevin) dynamics, which is based on the equations of motion for translations and rotations of the rigid filament under the influence of the external motor and thermal forces in the overdamped limit, i.e., neglecting inertia effects in comparison to frictional forces [6]. For micrometer-sized filaments, the overdamped limit is well justified. We determine (i) the translational equation of motion for the filament’s center-of-mass under the influence of forces arising from the attached molecular motors, thermal fluctuations, as well as hydrodynamic friction, and (ii) the corresponding rotational equation of motion for the filament’s orientation angle under the influence of the corresponding torques. The strength of the stochastic thermal forces and torques is taken to be proportional to temperature in accordance with the usual fluctuation-dissipation theorem (or Einstein relation), which guarantees that time averages correspond to thermodynamic averages [6]. In our simulation, these equations of Brownian dynamics are then integrated numerically [7], [8].

In each simulation time step, we first update the filament position and orientation according to the corresponding equation of motion. We then perform steps of the motor heads along the filament for the same time interval according to the motor force-velocity relation. Stepping of motor heads with surface anchored motor tails leads to forces that build up in the motor stalks that act as elastic springs. These forces are transmitted both onto the motor head affecting its stepping behavior and onto the filament to which the motor head is attached affecting the filament motion. Motor stalks equilibrate fast for given positions of the motor head and the anchoring point on the substrate. Therefore, motor stalk forces can be recalculated instantaneously after updating motor head or filament positions by applying the equilibrium force-extension relation of the motor stalk spring. We perform simulations by advancing motor head positions and filament position and orientation in discrete time steps 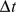 according to the forces transmitted by the stretched motor stalks. We also allow for stochastic attachment and detachment of motor heads during each time step. If not mentioned otherwise, we use the time step 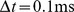. Values for motor parameters used in the simulations are summarized in Table 1.

**Table 1:** Values of motor parameters as used in the simulations.

Parameter	kinesin	myosin-V
Step size ℓ	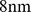 [9]	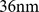 [10], [11]
Motor contour length *L_m_*	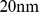 (trunc.) [1]	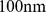 [13], [18]
Maximal velocity *ν*_0_	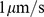 [26]	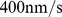 [10]
Motor stalk stiffness *K*	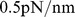 [14]	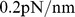 [12]
Motor stall force *F_s_*	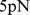 [25]	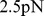 [10], [27]
Motor detachment force *F_d_*	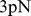 [29]	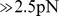 [27]
Detachment rate *k*_off,0_	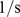 [29]	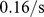 [30]
Attachment rate *k*_on,0_	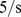 [34]	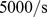 [35]

### Motor Proteins, Motor Stalks

**Figure 1:**
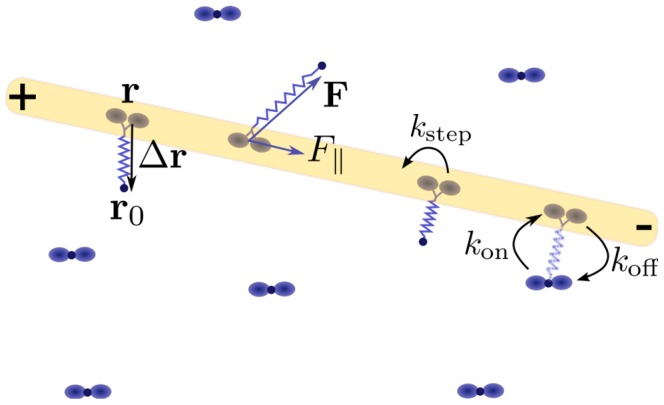
Top view of a gliding assay. When a motor head (blue dot) attaches to the filament (yellow rod) or steps along this filament, the motor stalk (blue spring) is stretched to an end-to-end vector 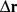. The force-extension relation of the motor stalk specifies a corresponding load force 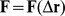 generated in the motor stalk. This stalk then acts as a spring which transmits this load force onto the filament. The component 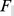 parallel to the filament also affects the motor head velocity.

Molecular motors are randomly distributed on the substrate surface with motor density 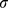. In simulations we mainly use 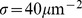. We use periodic boundary conditions to mimic a large substrate. Each molecular motor is described by two points: the position of its motor head and the position of its anchored motor tail, which are connected by the polymeric motor stalk. In a gliding assay filaments are pulled down to the substrate and glide at a constant small height, which has been determined for kinesin as 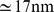 [16]. The quantity that varies during the motion of the motor head is a two-dimensional vector 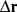, which is the projection of the vector pointing from the motor head to the anchored motor tail into the gliding plane, 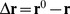, see Fig. 1, where 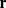 and 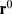 are the projected positions of motor head and anchored tail, respectively. The motor stalk is modeled as an elastic polymeric spring with a characteristic force-extension relation. For a small gliding height we can neglect the force component perpendicular to the substrate. The force-extension relation 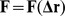 then specifies the two-dimensional force vector onto the motor head within the gliding plane, see Fig. 1.

There are a few experimental results on the elasticity of kinesin motor stalks [14]–[16]. The measurements of Ref. [15] on single-headed kinesin are consistent with a contour length 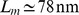 of the kinesin motor stalk. In Ref. [15] a fluctuation analysis of an attached cargo showed a non-linear spring behavior with a non-zero rest length for a single-headed kinesin. The stretching stiffness was measured as 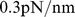, the compression stiffness as only 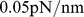. The gliding assay experiments of Ref. [16] with kinesin-1 showed that the gliding height of a MT is significantly less than the contour length and agrees with the mean square end-to-end distance of a freely jointed chain with 8 segments. In Ref. [14] direct optical trap measurements on a kinesin bead assay showed a linear force-extension with zero rest length relation for displacements parallel to the gliding plane and an elastic modulus around 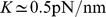. In the experiments of Leduc *et al.* in Ref. [1], truncated kinesins of contour length 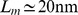 have been used. To allow comparison with the experimental results of Ref. [1] in the following, we will use this contour length for truncated kinesins and 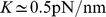 [14] in our motor stalk spring models (see Table 1).

For myosin-V, we use parameter values that have been reported for mouse myosin-V (see Table 1). For processive myosin-V motors the total contour length is 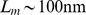 where the elastic extended tail domain has a length 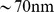 and the lever arm a length 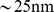 [13], [18]. Using optical traps a stiffness of 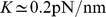 [12] for myosin-V has been measured.

We will compare different force-extension relations 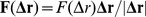 for motor stalk stretching in the gliding plane. Since the motor stalks are polypeptide chains, we will consider four generic models for polymers [17]:

(I,II) A *linear* relation.
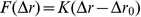
(1)corresponding to a harmonic or Hookian spring, which is characterized by a rest length 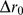 and the spring constant 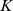 for the motor stalk. The contour length 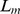 does not enter. It will be important to distinguish the two cases

(I) of a zero rest length 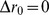 and

(II) a non-zero rest length 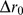. In the simulations we choose 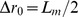.

The elastic coupling (I) also applies to linear force-extension relations 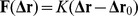 with a rest position 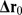: such a motor stalk corresponds to a stalk with a linear force-extension relation and zero rest length with a shifted motor tail anchoring point at 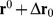.

(III) A *nonlinear* spring relation appropriate for a freely jointed chain (FJC) in three dimensions,
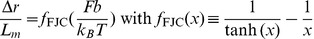
(2)which is characterized by the segment length 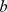 or the number 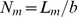 of flexibly connected segments in the motor stalk for given total length 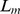 (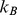 is the Boltzmann constant and 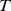 the temperature). Approximate inversion of (2) leads to [19], [20]

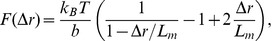
(3)the latter relation being equivalent to a linear entropic spring 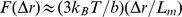 for small extensions 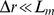, i.e., a relation of the type (1) with zero rest length 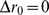 and spring constant 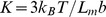. For kinesin we will use 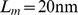 and 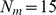, which gives in the linear small extension regime the same stiffness 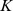 as used for the linear springs (I) and (II). For myosin-V we use 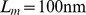 and 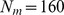 accordingly.

(IV) A *nonlinear* spring relation appropriate for an inextensible worm-like chain (WLC) with bending rigidity. We use the approximate relation as given by [21]

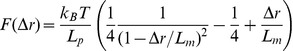
(4)which is characterized by the persistence length 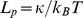 of the motor stalk and, thus, by its bending rigidity 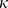. For kinesin we will use 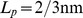 and 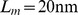, which gives in the linear small extension regime the same stiffness 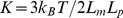 as used for the linear springs (I) and (II). For myosin-V we use 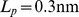 and 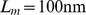.

Note that the zero rest length for the springs (I),(III), and (IV) only refers to the projected displacements in the gliding plane. The total displacement including the height coordinate can still exhibit a non-zero rest length: for a filament in the gliding plane, the interaction forces between the filament and the substrate surface have to be balanced by the perpendicular force components arising from the motor stalks.

### Filament Dynamics

The filament is taken to move within the quasi-two-dimensional gliding plane at approximately constant gliding height. Thus, we ignore the surface roughness of the underlying substrate surface. The rigid filament has then two degrees of freedom, its center of mass position 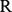 and its orientation angle 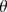. The MT has a diameter of 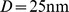 and we use a length of 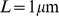 in our simulations. This choice is motivated by the experiments of Leduc *et al.* [1], where relatively short MTs are studied as well. F-actin has a diameter of 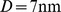, and we use lengths 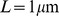 or 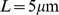. As mentioned before, the filament motion in the two-dimensional gliding plane is simulated by Brownian dynamics, i.e., we solve the overdamped equations of motion (5) for the center of mass 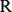 and (6) for the orientation angle 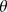.

If 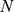 motor heads are attached to the filament with motor head positions 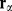 and fixed motor tail positions 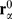 (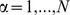), each attached motor head transmits the stretching force 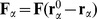 of the motor stalk and a corresponding torque 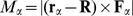 onto the filament. The overdamped equations of motion for the filament’s center-of-mass 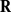 as given by
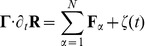
(5)and for the orientation angle 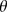, which has the form
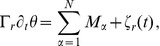
(6)contain the motor forces 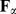 and motor torques 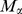, the thermal forces 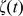 and thermal torques 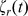, as well as the frictional forces and torques on the left hand side of these two equations. The frictional forces are characterized by the matrix 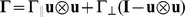 depending on the translational friction coefficients 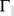 and 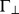 as well as on the unit vector 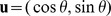 for the filament orientation. The rotational friction coefficient is denoted by 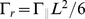. Friction coefficients are given by 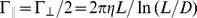, where 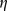 is the viscosity of water. Using a higher viscosity 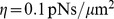 in the simulation, we have a friction coefficient of 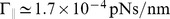 for MTs and 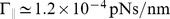 for F-actin of 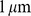 length.

### Motor Stepping, Attachment, and Detachment

The stretching force 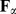 is also transmitted onto the motor head 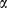. We assume that only the component 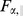 parallel to the filament has an effect on motor velocity, whereas the component 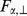 perpendicular to the filament can be neglected [22]. The velocity of motors walking along a filament decreases monotonically from a maximal velocity 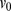 without external load to zero at the stall force 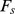 [23]–[25]. We approximate the relation between the force 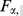 parallel to the filament and the mean velocity 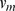 of the molecular motor by a piecewise linear function, which is justified by experimental results for kinesin [26]. In the piecewise linear force-velocity relation resisting forces 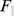 slow down motors linearly,
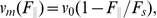
(7)whereas motors move with the maximal velocity 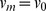 for assisting forces. The stall force 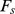 is taken to be 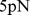 for kinesin [25]. For the zero force velocity of kinesin, we use the value 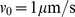, which applies to ATP concentrations that exceed 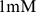 [26] (see Table 1). Note that the experiments of Leduc *et al.* [1] were performed at much lower ATP concentrations in order to reduce the stepping frequency of motors and, thus, improve step detection. The experimental motor velocities observed in Ref. [1] are only of the order of nm/s. For processive myosin-V motors, we will use the same piecewise linear force-velocity relation with a stall force 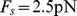 [10], [27] and a maximal motor velocity 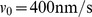 [10] (see Table 1). These parameter values from Refs. [10], [27] are for chicken brain myosin-V but very similar values 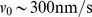 and a similar force-dependence of the kinetics have been reported for mouse myosin-V [12].

In Refs. [4], [5] we approximated the motion of the motor head on the filament as a continuous deterministic motion with velocity 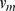, which makes the observation of MT stepping impossible. Here, we employ a realistic model with discrete stochastic motor steps at a force-dependent stepping rate 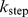. Kinesin moves along MTs towards the plus end with a discrete step size of 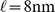, which is the size of a tubulin dimer [9]. Myosin-V moves along actin filaments with a step size of 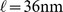 [10], [11] towards the barbed end. The force-dependent mean velocity 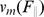 as given by (7) is the result of discrete stochastic motor steps with step size 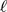 and the stepping rate 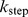. In order to obtain the same mean velocity, this stepping rate has to be force-dependent and chosen as
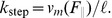
(8)


We simulate the motion of molecular motors with a stochastic stepping mechanism which means that molecular motors move by a discrete step 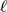 during the time interval 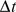 with a probability 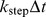 or remain at their position with probability 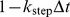. As mentioned before, we do not resolve the two heads of the double-headed motors.

Because of the force dependence of the stepping rate, the order, in which the different motors perform a step, depends on the loading state of their motor stalks. Motors which are pulled backwards have a smaller stepping probability, motors which are pulled forward are more likely to move. We assume a fixed motor step size 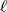 [28], which is independent of the load force. The variance in experimentally obtained step size distributions [26] appears to be force-independent and can be attributed to noise in the measurement process.

We will also use a more refined stochastic modelling for the detachment and attachment of motor heads from and to filaments as compared to the model employed in Refs. [4], [5]. The detachment process of a motor head from the filament is a force-dependent stochastic process and the detachment rate 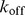 is given by 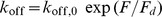 where 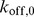 is the detachment rate in the absence of force and 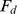 is the detachment force. For kinesin, we choose the values 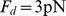 [29] and 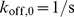 [29]. For myosin-V, we use 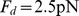 equal to the stall force because the detachment appears force-independent in experiments [27] and 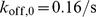 [30] (see Table 1).

**Figure 2:**
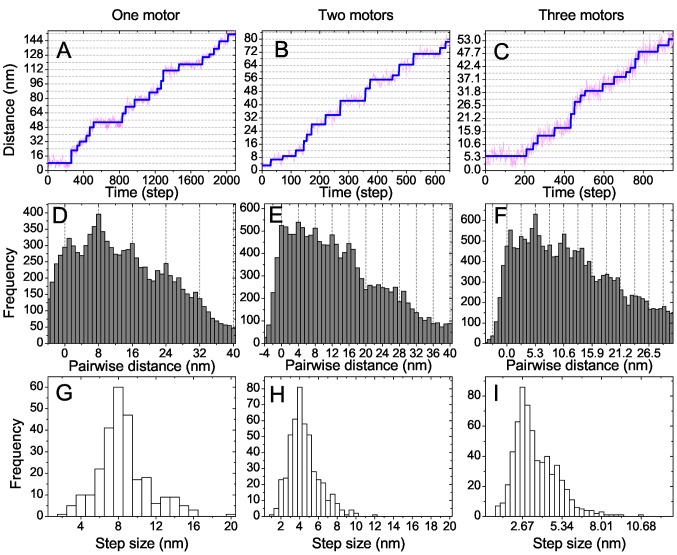
MT stepping behavior for a linear force-extension relation (1) of motor stalks with zero rest length (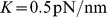). Top row: Walked distances (in nm) of the MT center of mass as a function of time (in simulation steps 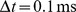) and best fit result of the step detection algorithm. Middle row: Histograms of pairwise distances. Bottom row: Step size distributions. A,D,G: One motor (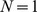), B,E,H: two motors (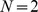), C,F,I: three motors (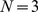) are attached to the MT. The step size distributions exhibit peaks at 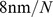.

The attachment of a motor to the filament also represents a stochastic process depending on the force-extension relation of the polymeric motor stalk, which gives rise to the potential energy 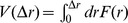 for the motor head position relative to the fixed motor tail position. We assume fast orientation of the motor heads to the filament orientation during the attachment process. The potential energy 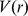 determines the on-rate 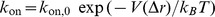 for motor head attachment at a distance 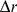 from the motor tail position. The on-rate is thus decreased by the stretching energy, which is involved in the binding process of the motor-head. If we assumed that an identical reaction coordinate 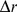 could be used for attachment and detachment of motor heads, detailed balance would require the on-rate 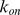 to contain an additional factor 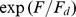 involving the detachment force. This has been pointed out in Refs. [31], [32]. We argue that attachment and detachment of motor heads proceeds along different pathways: whereas the detachment process of the motor head always starts with the motor stalk in a strained configuration and the distance 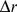 between motor head and anchored tail can serve as reaction coordinate, the attachment process starts from a relaxed configuration of the motor stalk and can proceed along many different paths in the configurational space of the motor stalk. As a consequence, unbinding and rebinding of motor heads cannot be described by the same reaction coordinates [33], and we can use the simple expression 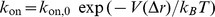 for the motor head attachment rate. We also note that inclusion of an additional factor 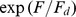 into 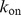 would have a negligible effect on our results as we checked explicitly. We use 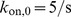 for the 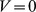 on-rate for kinesin [34] and 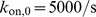 for myosin-V [35] (see Table 1). Using the additional Boltzmann factor we assume that polymeric motor stalks have a sufficiently fast dynamics such that the equilibrium force-extension relation is always satisfied during the attachment process. As a result of this attachment modelling, the motor attachment radius around the MT is roughly given by the distance 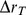, where the motor stalk deformation energy becomes of the order of the thermal energy, 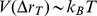. For most of our analysis of the apparent fractional filament steps, the attachment and detachment processes are not crucial because we analyze the filament trajectories for a *fixed* number of attached motors 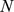, i.e., *between* motor attachment or detachment events, see Figs. 2(A,B,C). We also perform simulations keeping the *average* number 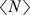 fixed. In the latter case, the attachment process is important because it determines the typical attachment length 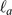 of motors, which is the distance over which attachment of a motor is probable. The attachment length is approximately given by the motor stalk extension corresponding to the thermal stretching energy, 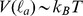.

### Filament Stepping Analysis

In the simulations we analyze the stepping motion of filaments by detecting the number 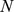 of motors to which the filament is attached during transport and analyzing the stepping motion for each number of attached motors 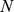 separately by measuring three quantities.

First, we record trajectories of the filament center of mass and determine the *walked distances* 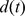 of the filament center of mass along its trajectory as a function of time. Steps in filament motion give rise to steps in the walked distance curves 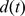. This procedure is analogous to the experimental procedure of Leduc *et al.* [1].

Secondly, we calculate *histograms of pairwise distances* along the filament trajectories. From the walked distances 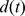, pairwise distances 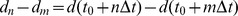 are calculated for 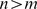 for a fixed reference time 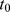. All these pairwise distances 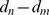 are collected in a histogram. Peaks in the distribution of pairwise distances signal steps in filament motion: If there is a well-defined filament step length peak positions should occur at multiples of this filament step length. This procedure is identical to the analysis of the corresponding experimental data by Leduc *et al.* [1].

Finally, we use the model-independent step finding algorithm described in Ref. [36] to obtain step size distributions from the walked distances 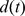 of filaments. Also these results can be directly compared to the experimental data of Ref. [1] (where a different step finding algorithm was used). We measure filament step size distributions not only for a fixed number 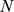 of attached motors as in Ref. [1] but also for the experimentally more accessible situation of a fixed *average* number 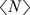 as determined by the surface density (or coverage) of the motors on the substrate surface.

## Results

### Fractional MT Stepping in Kinesin Assays

First, we study fractional steps of MTs in a kinesin gliding assay. The simplest model for kinesin motor stalk elasticity is a linear spring model of the form (1) with a *zero* rest length 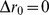. This model is independent of the motor contour length 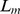 and the force is not diverging such that motors will not detach if extensions 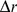 exceed the contour length 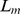 of the motor stalk. To overcome this problem, we let all molecular motors detach from the filament when the length extensions reach their contour length.

In the simulation motor stepping leads to a similar stochastic “stepping” motion for MTs, and the step size depends on the number of molecular motors attached on the filament as shown in the walked distances in Figs.2(A,B,C) and histograms of pairwise distances in Figs. 2(D,E,F). If transported by a single kinesin MTs walk on the substrate surface with a step size of 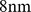 equal to the motor step size (Fig. 2(A)). Each time, the attached molecular motor takes one 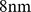 step on the MT, the motor stalk is extended, which generates a force in the motor stalk pulling the MT in the opposite direction. When the MT has moved by 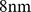 driven by this force the motor stalk is relaxed and the overdamped MT motion stops because of a short relaxation time 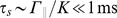.

**Figure 3:**
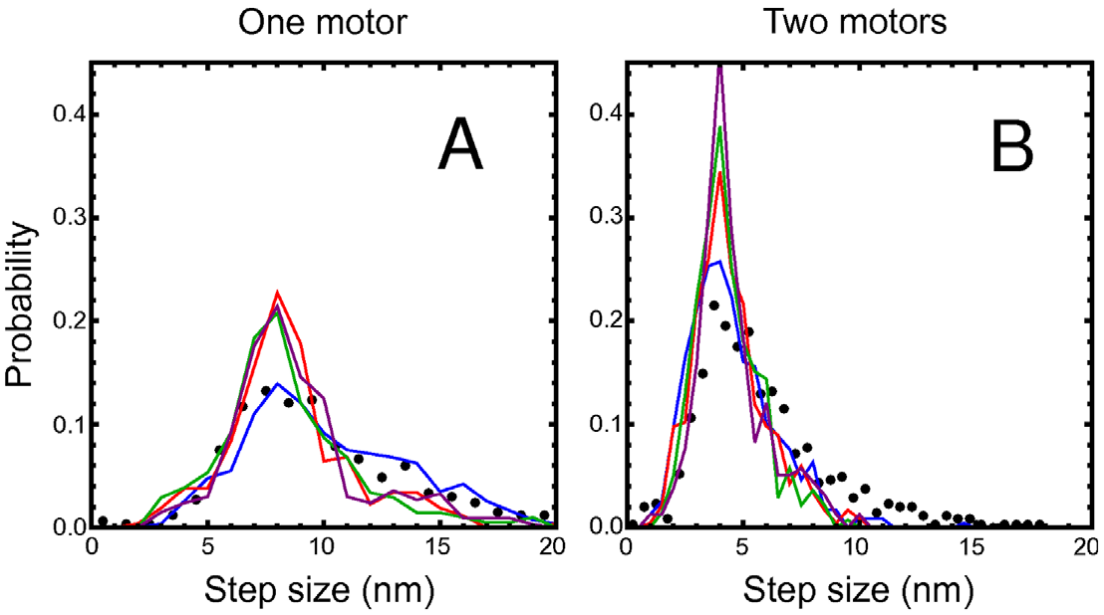
Normalized step size distributions for (A) 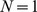 and (B) 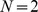. Experimental data from Ref. [1] shown as black point, simulation data as lines. Red line: Motor stalk that acts a linear spring (I) with zero rest length. Blue line: linear spring (II) with non-zero rest length. Green line: freely jointed chain (III). Purple line: worm-like chain (IV).

MT steps of 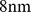 are also dominant in the histogram of pairwise distances for single motor transport in Fig. 2(D) and in the step size distribution obtained with the step detection algorithm in Fig. 2(G). Only if the stochastic waiting times between successive stochastic motor steps are short the step finding algorithm interprets such steps as 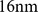 double-steps. In the experiments of Ref. [1] double-steps appear more frequently because of the additional noise from the MT position measurements by quantum dot position tracking. Therefore, the experimental step size distribution for 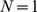 has more weight around a 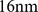 peak. The slightly higher noise level in the experiment also gives rise to a broadening of the step size distribution around the dominant 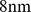 and the smaller 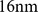 peak. Otherwise there is quantitative agreement between the experimental step size distribution of Ref. [1] and our simulation result as can be seen in Fig. 3(A).

We also find fractional 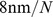 steps in MT motion for transport by 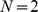 or 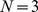 motors (Figs. 2(B,C)), as can also be seen by the peak positions in the histogram of pairwise distances (Figs. 2(E,F)) and clearly in the step size distributions (Figs. 2(H,I)), which are centered around 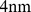 for 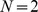 and 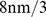 for 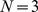. Also the step size distribution for 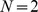 quantitatively agrees with the experimentally observed step size distribution, see Fig. 3(B). As for 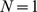, the experimental step size distribution is slightly broader because of the additional noise from the MT position measurement.

**Figure 4:**
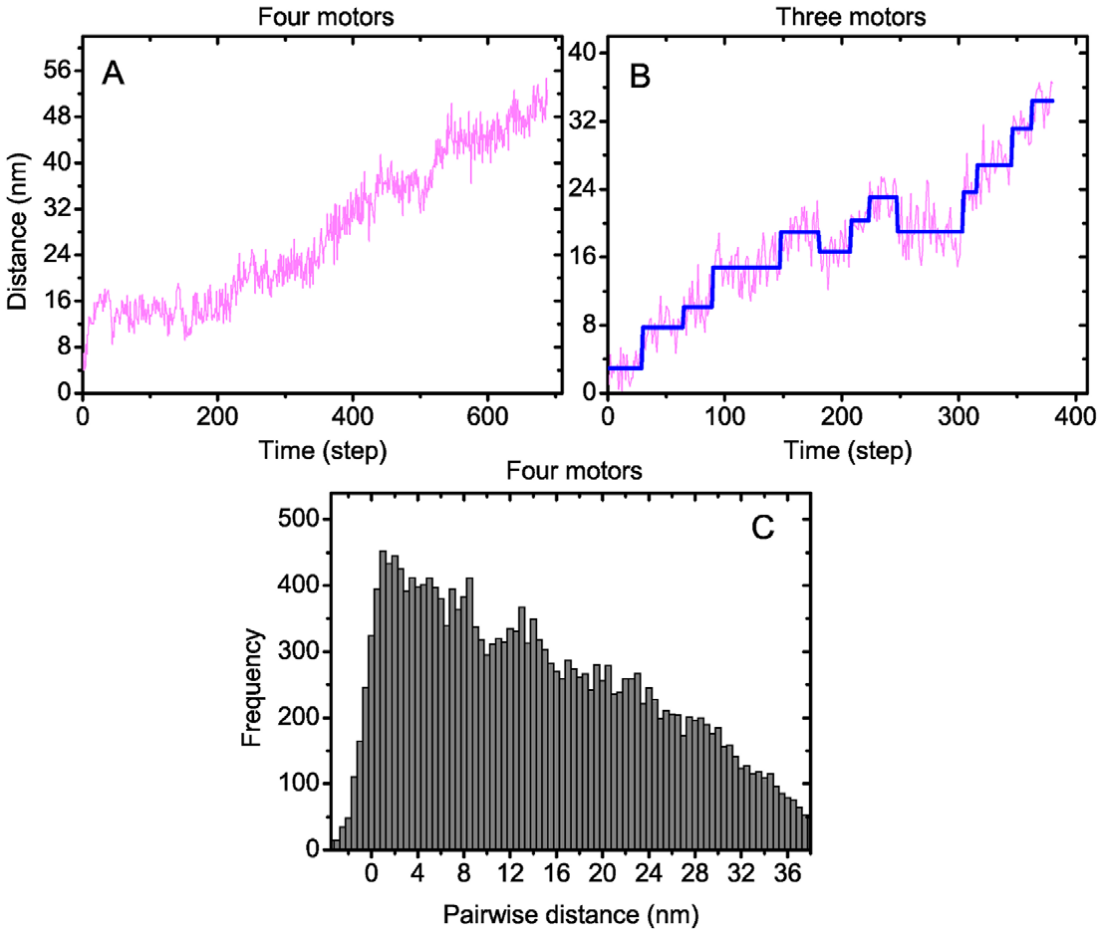
MT stepping behavior for 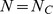. (A,B) Walked distances (in nm) of the MT center of mass as a function of time (in simulation steps 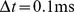); (A) Motor stalks that act as a linear spring (I) with zero rest length and motor number 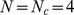: the step finding algorithm fails to identify steps; (B) linear springs (II) with non-zero rest length and 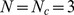: typical step sizes are significantly larger than 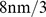. (C) Histogram of pairwise distances for linear springs (I) with zero rest length and 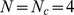. There are no clear peaks.

Smaller steps for 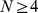 cannot be observed because the thermal fluctuations of the MT position are too large. This results in a failure of the step finding algorithm to identify steps for 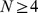. This is illustrated by the walked distances for 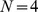 shown in Fig. 4(A) and the histogram of pairwise distances, Fig. 4(C), which does not exhibit clear peaks.

### Thermal Fluctuations Limit Observable Fractional Step Sizes

Thermal noise limits the observability of fractional steps. In our simulations, the thermal noise level at room temperature is too high to observe even smaller fractional 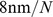 steps with 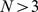 as demonstrated in Fig. 4(A,C). If thermal fluctuations of the MT about its mean position between motor steps become larger than half the step size, MT positions before and after a step “overlap”, and step finding algorithms can no longer identify a MT step. In thermal equilibrium, equipartition gives MT position fluctuations 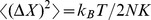 for 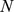 attached motors by harmonic motor stalks with spring constant 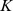. We consider only one component 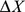 along the MT orientation. This results in typical MT positional fluctuations of the order of 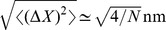 for simulation parameters (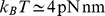). It becomes difficult to distinguish fractional steps of size 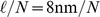 from thermal fluctuations if fluctuating positions before and after the step overlap. This leads to the condition 
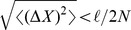
 for the observation of fractional steps. Because 
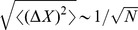
, this condition is equivalent to the inequality
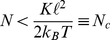
(9)for the motor number 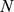, which defines the critical motor number 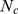. For the parameters of the MT/kinesin system as used in our simulations, the critical number 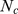 as given by (9) becomes 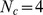 in agreement with our simulation results. Experimentally, already larger fractional step sizes such as 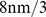 could be unobservable because of the additional noise from the MT position measurements. Therefore, 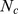 only represents an upper limit for the observability of fractional steps set solely by thermal fluctuations. Assuming an additional experimental noise level 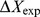 for the MT position measurement, which is independent of thermal fluctuations, we can formulate a criterion for observable fractional steps as 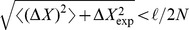 which leads to



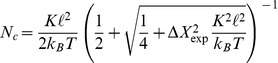
(10)For a realistic experimental noise of 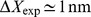 and with the same parameter used in the simulation, this leads to 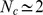 as observed in experiments.

The derivation of the critical motor number (9) did also not include possible effects from non-linear stalk elasticity. It should be applicable as long as these effects are small, which is the case for step sizes 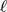 much smaller than motor contour lengths 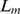. This is fulfilled for the gliding assays considered here as will be discussed in more detail below.

It is important to note that 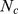 depends quadratically on the motor step size 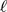 according to (9). Therefore, we can expect to observe a much higher 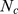, i.e., much smaller fractions of full steps for myosin-V motors, which have a 4–5 fold larger step size of 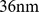 as compared to kinesin, as long as the motor stiffness 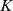 is not much smaller. We will discuss this point below.

### Force Equilibrium Model

Motor stepping is much slower than the equilibration dynamics of the motor stalks and the filament position: For MTs the maximal motor speed 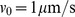 roughly corresponds to 1 step per 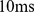, whereas the typical filament position relaxation time is 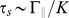, which is well below 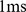. Therefore, mechanical equilibrium of the filament position can be reached after each motor step, and our results can be rationalized by a simplified force equilibrium model.

In the force equilibrium model we consider a filament with 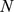 motors attached with initial motor stalk extensions 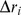 (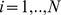), which are the result of previous motor steps, and take the 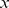-coordinate parallel to its orientation. Then, the 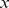-component 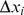 of a motor stalk extension vector is changed by a step size 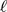 in a single step of one of the motors. It is assumed that after each motor step the filament center of mass 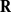 adjusts quickly by moving its center of mass by 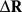 in order to relax the motor stalk stretching forces in 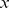
- and 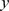-direction. Displacing the filament center of mass by 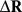 leads to new motor stalk extensions 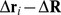, such that the new equilibrium 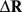 is determined by
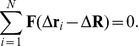
(11)


We assume that filament rotation is slower, which is justified for sufficiently long filaments because 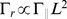, and neglect rotational motion towards torque equilibrium.

The force equilibrium model explains that, in the absence of thermal noise, fractional 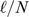-steps are an intrinsic feature of the elastic coupling (I), characterized by a *linear* force-extension relation with *zero* rest length, and should be observable for all 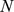. For the spring (I), i.e., a linear force-extension relation (1) with zero rest length, the force equilibrium in the 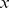-direction parallel to the filament decouples from the force equilibrium in the perpendicular 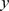-direction. For motor stalk extensions 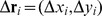 and filament displacement 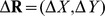, the parallel force equilibrium (11) for the spring (I) gives a linear equation for the equilibrium filament displacement 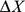,
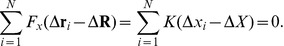
(12)The resulting equilibrium displacement
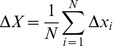
(13)is independent of the spring stiffness 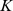. The perpendicular filament displacement 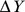 decouples from the parallel force equilibrium (12) and the parallel displacement 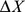 and can be determined from the perpendicular force equilibrium.

**Figure 5:**
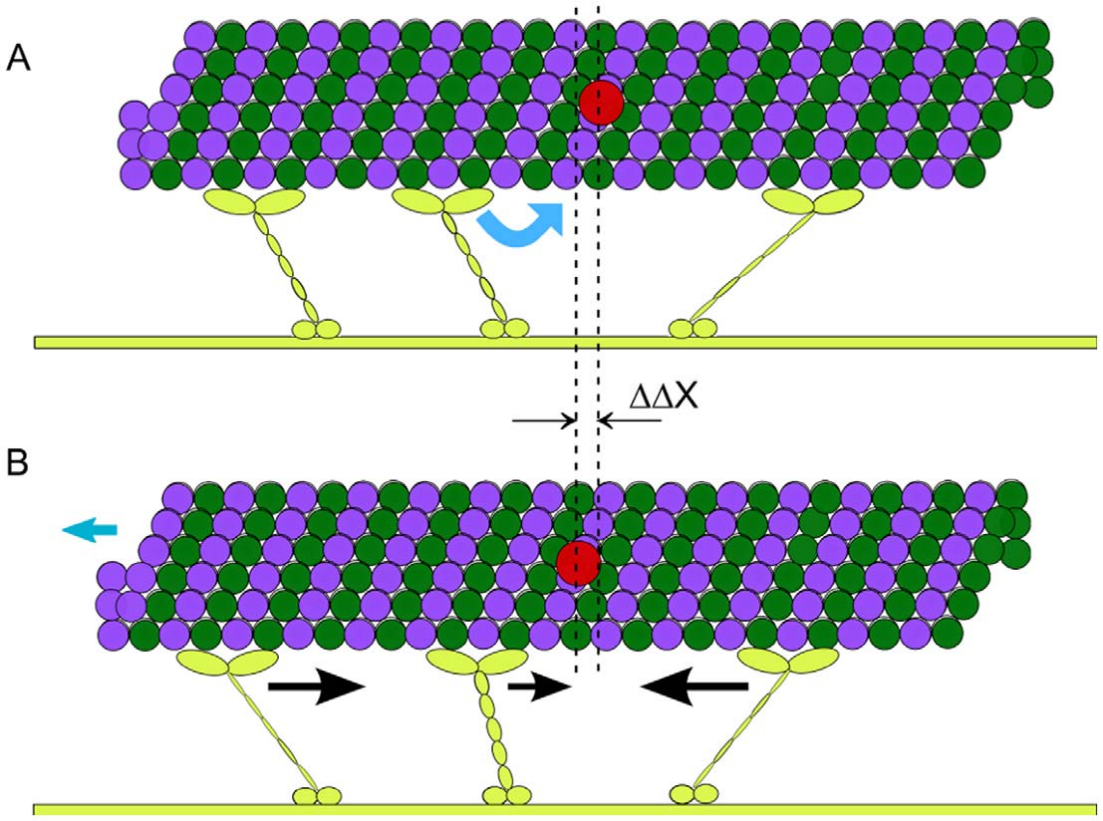
Force equilibrium model. After one of the attached motors moves one step 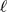 (A) the mechanical equilibrium position of the MT is shifted by 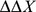 to establish force equilibrium (B).

If one of the attached motors moves one step 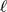 in 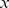-direction along the filament, we have 
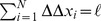
, which leads to a change
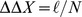
(14)of the filament position in the new mechanical equilibrium as illustrated in Fig. 5. In particular, this change of the filament position is independent of the initial motor positions 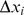 and, thus, from the load on each motor and the order of motor stepping for 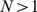. Therefore, we expect to observe a *unique* apparent filament step size 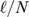. This argument is valid for arbitrary 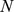 such that in the absence of noise, *all* fractional filament step sizes 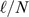 with 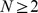 would be observable for a stalk, which behaves as the linear spring (I).

In simulations and experiments, we do not observe a sharp step size distribution for the linear spring (I) because of the additional thermal noise and, in the experiments, also because of noise from the filament position measurement. Such noise can be included in the force equilibrium (11) as additional, approximately Gaussian random forces. If the time interval between consecutive filament steps is short, steps can be missed by the step finding algorithm resulting in the detection of a double step instead of two consecutive single steps. This effect leads to a distortion of the step size distribution since a certain fraction 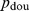 of single steps is counted as double steps. For the linear spring (I), both noise and double step detection combine in the force equilibrium model to give double- (or even multiple) Gaussian distributions consisting of a superposition of Gaussians centered around multiples of 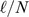 both for 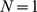 and 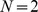 as observed experimentally Ref. [1] and in our simulations. Deviations from such double-Gaussian distributions indicate deviations from a linear motor stalk elasticity with zero rest length. In the theoretical description used here, the ATP concentration enters only via the force-velocity relationship (7), which involves two parameters, the zero-force velocity 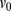 and the stall force 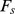. In the present study, we focused on relatively high ATP concentrations that exceed 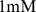, which implies the value 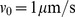 for the zero-force velocity. Furthermore, the stall force 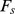, which was chosen here to be 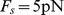, depends only weakly on the ATP concentrations as experimentally observed in [25], [26]. An increase in the motor velocity and, thus, the stepping frequency will slightly increase the fraction 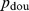 of false double step detections by the step finding algorithm, which increases the peak around 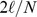 relative to the peak around 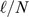 in the filament step size distribution. An increase in the stall force has only negligible effects on the filament step size distribution.

### Influence of Stalk Elasticity on MT Stepping

The mechanical force equilibrium that is reached after one attached motor performed a step depends on the the number of motors attached to the filament and the elastic properties of the motor stalks. Therefore, using microscopic Brownian dynamics, we compare the influence of the different force-extension relations, a linear relation with (I) zero and (II) non-zero rest length, (III) a freely jointed chain relation, and (IV) a worm-like chain relation on the stepping motion of the MTs.

#### Non-zero rest length

**Figure 6:**
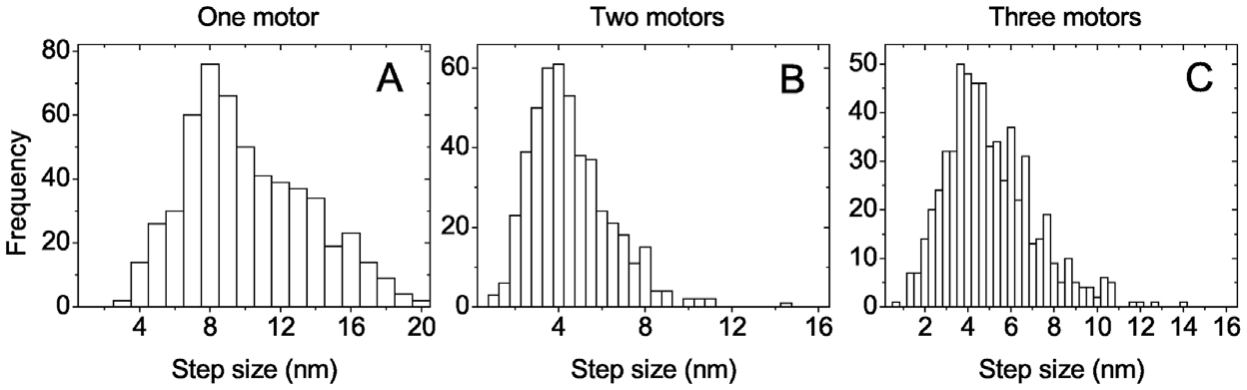
MT step size distribution for a linear force-extension relation (1) of motor stalks with non-zero rest length (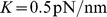, 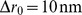). A: One motor (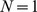), B: two motors (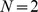), C: three motors (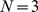). For three motors, the peak is at a value significantly larger than 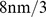 with a broad step size distribution.

The linear spring (II) with *non-zero* rest length 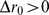 appears, at first sight, to be not much different from the linear spring (I) with zero rest length. Somewhat surprisingly, a non-zero rest length can, however, lead to a rather different MT stepping behavior. For non-zero rest length, we still observe 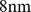 MT steps if one motor is attached but the step size distribution is significantly broadened as compared to zero rest length, see Fig. 6(A). Similarly, for MT transport by 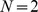 motors (Fig. 6(B)), the step size distribution exhibits peaks around 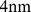 but is considerably broader as for a zero rest length. For three motor transport, on the other hand, the peak in the step size distribution is at a value larger than 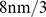 with a broad distribution such that fraction third steps cannot be observed for non-zero rest lengths, see Fig. 6(C).

These simulation results for the linear spring (II) can be rationalized in the framework of the force equilibrium model. For 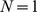 all MT displacements 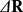 with 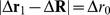, i.e., on a ring of radius 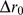 around 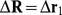 are solutions of the force equilibrium (11). This degeneracy corresponds to a soft mode in the actual simulation dynamics with the motor stalk and the attached MT rotating around the anchoring point resulting in large diffusive displacement fluctuations, which broaden the step size distribution for 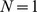 considerably. For 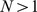 and a non-zero rest-length, the force equilibrium of several motor stalks results in two *coupled and non-linear* equations for the displacement vector 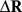,
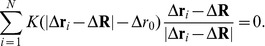
(15)


The force equilibrium is at displacements 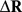, which are close to all 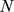 circles 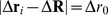. For attachment distances shorter or comparable to the rest length 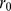, the coupled non-linear eqs. (15) give rise to a strong coupling between parallel filament motion 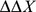 and perpendicular motion 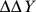 resulting in a filament stepping not aligned with filament orientation and broadening of the filament step size distribution. For 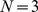, the broadening of the step size distribution shifts the peak in the step size distribution to a value significantly larger than 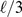, see Fig. 6 (C) such that fractional steps with 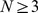 cannot be observed and 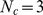 for this model. This is also evident from the walked distances shown in Fig. 4(B) for 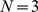. The absence of a sideways motion not aligned with filament orientation in experiments [1] favors motor stalk models with zero rest length in the gliding plane.

#### Non-linear force extension relation

Finally, we investigated whether an intrinsically non-linear force-extension relation of motor stalks as described by the freely jointed chain (III) or the worm-like chain (IV) gives rise to similar effects in the step size distributions. The comparison of normalized step size distributions in Fig. 3 for all four motor stalk models clearly shows that the effects arising from the non-linearities of freely jointed chains (III) and worm-like chains (IV) are relatively small and hardly change the behavior observed for linear springs (I).

To explain this result we note that, for parameter values corresponding to the same motor stalk contour length and linear spring constant at small extensions, both freely jointed chains and worm-like chains are well in their *linear* regime at energies around the thermal energy 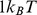. The thermal energy is the typical stretching energy if motor attachment is governed by a on rate 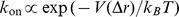 with a Boltzmann factor containing the stretching energy. Also an additional displacement by one motor step 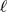 does not lead to non-linear effects as long as 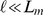, which is fulfilled both for kinesin and myosin-V. Because both freely jointed chains and worm-like chains also have a zero rest length, they behave very similar to the linear harmonic model with zero rest length.

The comparison of simulated normalized step size distributions in Fig. 3 for all four motor stalk models show that a non-zero rest length has the most pronounced effect and results in a significant broadening of step size distributions, whereas intrinsic non-linearities as described by freely jointed chains or the worm-like chains deviate only little from the linear model with zero rest length.

Fig. 3 also shows the normalized experimental step size distributions from Ref. [1] for comparison. The linear spring (II) with non-zero rest length fits the experimental data best but one has to keep in mind that there is additional noise from the MT position determination in the experiment. Such additional noise will also give rise to a lowering of the peaks at 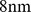 and 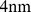, respectively, and a broadening of the step size distributions. Therefore, the linear spring (I) and the non-linear springs (III) and (IV) with zero rest lengths can lead to equally good fits if additional noise is applied in the simulations, and it is difficult to draw definite conclusions about the stalk elasticity from the comparison of experimental and simulation data for 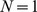 and 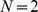, The situation is much more conclusive for 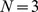, for which a non-zero rest-length of the motor stalk clearly shifts the peak of the step size distribution to values larger than 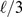 (Fig. 6 (C)) in our simulations. However, for 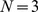, experimental step size distributions are not available so far.

### Fractional F-actin Stepping in Myosin-V Assays

**Figure 7:**
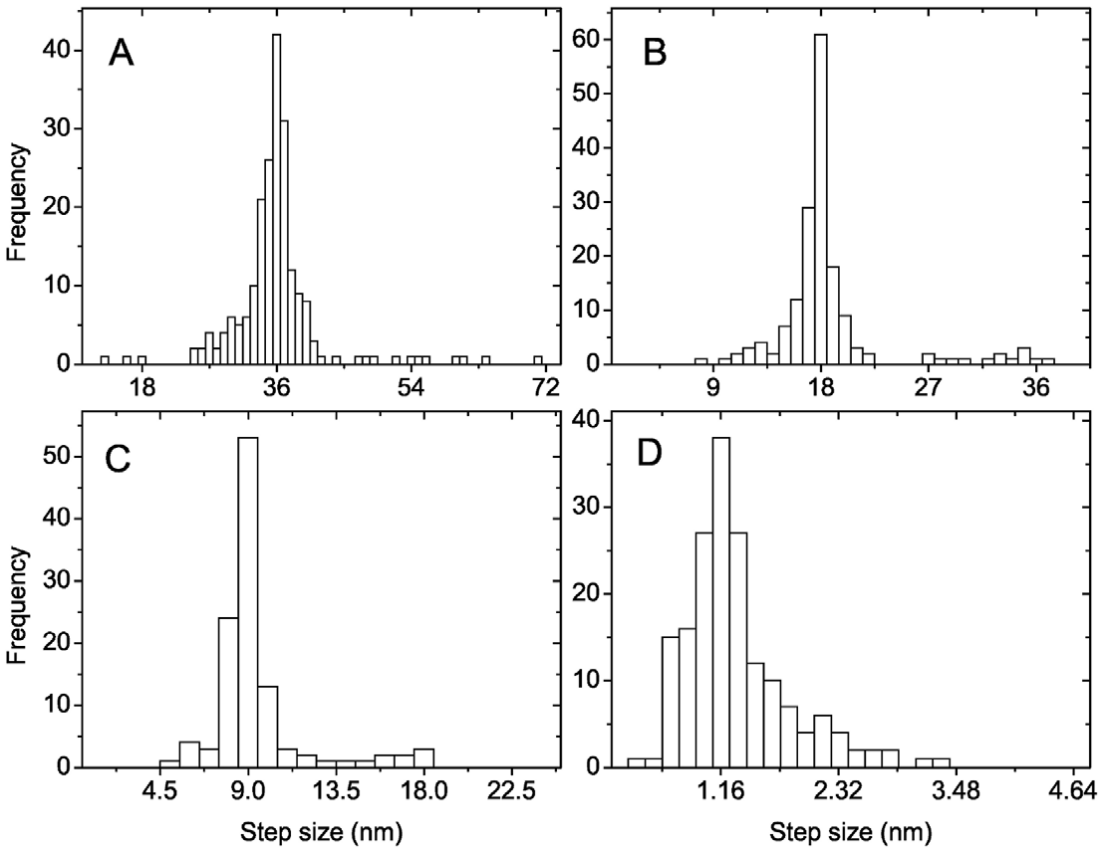
F-actin step size distributions in myosin-V gliding assays for fixed motor number 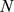. (A) 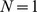; (B) 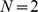; (C) 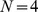; and (D) 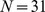. Results are obtained for a linear force-extension relation (1) of motor stalks with zero rest length (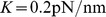). The peaks are at step sizes 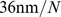 with 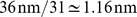.

We also simulated gliding assays consisting of actin filaments and myosin-V motors, for which fractional filament steps have not been studied experimentally so far. This system is interesting because of the much larger step size 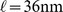 of myosin-V. We focused on motor stalks that act as linear springs (I) with zero rest length. Because the critical motor number 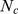 below which fractional steps of size 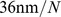 should be observable increases quadratically with the step size 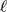 according to (9), we can predict that much smaller fractions of full steps should be observable for myosin-V. For 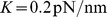, we find 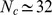 based on our criterion (9). This is confirmed by our simulations where we observe fractional filament steps up to 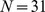 corresponding to a step size 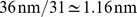, see Fig. 7. In the presence of additional experimental noise 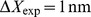 the corresponding criterion (10) predicts that fractional steps up to 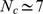 should also be experimentally observable.

The critical number 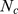 of observable fractional filament step sizes is also inversely proportional to the stiffness 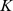 according to eq. (9). For softer motor stalk stiffnesses we therefore expect to observe much less fractional step sizes. To test this dependence we also performed simulations for a reduced stiffness 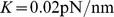. Indeed, we can only observe half-steps corresponding to 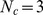 in the simulations, which agrees again with criterion (9). This shows that an experimental determination of 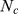 for a myosin-V gliding assay can give information about the motor stalk stiffness of myosin-V.

So far, we have implicitly assumed that the overall filament trajectories can be decomposed into distinct segments, each of which is characterized by a fixed motor number 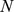. Such a decomposition is always possible in simulations and has also been achieved experimentally in Ref. [1] up to 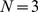. However, it is hardly possible to experimentally distinguish segments with 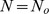 from those with 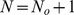 for large values of 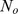.

In contrast, the *average* number 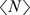 of motors that actively pull on the filament can be directly controlled experimentally via the surface density (or coverage) 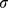 of the motors on the substrate surface. For a filament of length 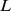, this average number is given by 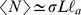 [5] and is, thus, proportional to the motor surface density 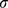, the filament length 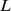, and the attachment length 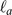 of the motors, the latter being approximately equal to the motor stalk extension arising from thermal fluctuations, which implies 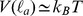.

**Figure 8:**
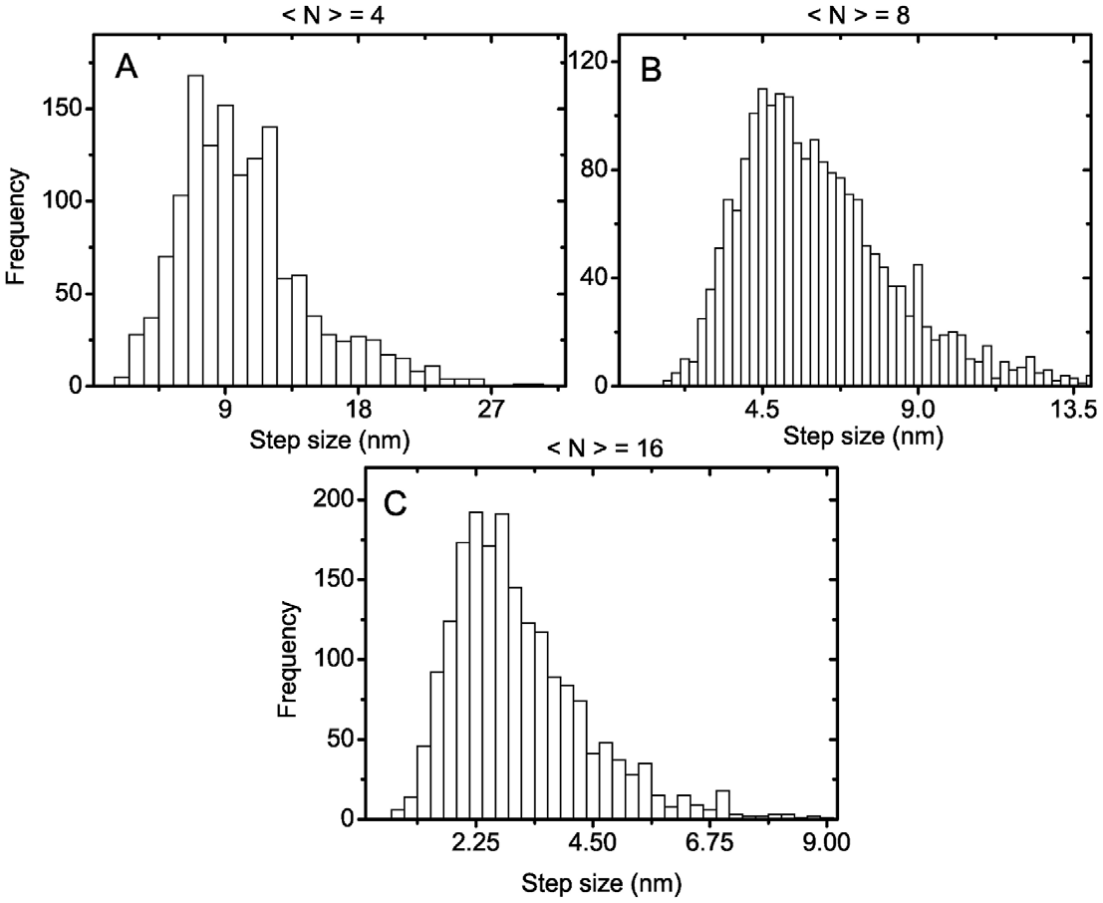
F-actin step size distributions in myosin-V gliding assays for fixed average motor number 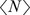. (A) 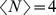; (B) 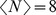; (C) 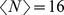. Results are obtained for a linear force-extension relation (1) of motor stalks with zero rest length (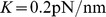). The peaks of the distributions are located at 7 nm, 4.5 nm, and 2.25 nm for 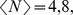 and 16, respectively.

Thus, we performed simulations for actin/myosin-V gliding assays, for which the motor density has been adjusted to produce a certain average number 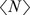 of the attached motors whereas the actual motor number 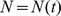 becomes time-dependent and fluctuates around its average value 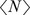. Examples for the corresponding distributions of the filament step size are shown in Fig. 8. Inspection of this figure reveals pronounced peaks in the step size distributions, with a decreasing peak position as a function of the average filament number 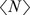. In comparison to simulations with fixed motor number 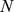, the filament step size distribution for fixed 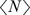 is broadened and its peak position is shifted to values that are slightly smaller than 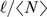. This can be understood as follows. The step size distribution for fixed average 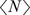 is a superposition of different, approximately Gaussian distributions corresponding to the different values of 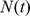. For 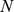 attached motors with harmonic motor stalks with spring constant 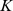, the step size distribution is a Gaussian centered around 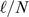 with a width given by 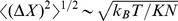, which decreases for increasing 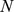. The superposition of these Gaussian step size distributions gives rise to a broadening of the step size distribution. The decreasing width of the superimposed Gaussians for higher values of 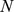 gives rise to a shift in the peak step size of the superposition to a value smaller than 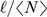. In any case, our simulations show that filament step size distributions for fixed average motor number 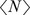 also exhibit peaks at fractional step sizes over a wide range of 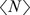-values with 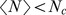.

## Discussion

We have investigated fractional filament steps 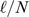 in gliding assays both for MTs and F-actin, which are transported cooperatively by a number 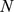 of kinesin or myosin-V motor proteins with different step lengths 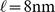 and 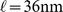, respectively. Our simulation data shows that the filament stepping behavior crucially depends on the number 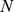 of transporting motors and the elasticity of the motor stalks, which transmit forces onto the filaments. We have employed four different elastic elements to describe the motor stalks in our simulations: linear springs with zero and non-zero rest length as well as freely jointed chains and worm-like chains.

For the kinesin gliding assays we found filament step size distributions and histograms of pairwise distances for transport by 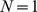 and 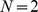 motors (Figs. 2(D,E,G,H)), which show quantitative agreement with the experimental results of Ref. [1] using a linear motor stalk elasticity with zero rest length. Small differences arise from the somewhat higher noise levels in experiments, which is generated during the MT position measurement. This demonstrates that our simulation model, which does not include any motor coordination mechanism apart from a coordination via the load force distribution, is able to quantitatively reproduce the experimental results of Ref. [1] on the resulting step-like transport of microtubules by kinesin.

Thermal fluctuations and additional experimental noise limit the observability of small fractional steps. We derived the criterion (9) that fractional steps are only observable for 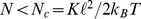 for a linear motor stalk elasticity with zero rest length and for purely thermal noise as in our simulations. This analytical estimate gives 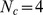 for the kinesin-assay in agreement with our simulation results, where fractional steps can be observed up to 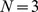 (Figs. 2(F,I)). In experiments, the value 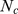 can be smaller because of additional noise from the filament position measurements, see eq. (10).

In our simulations, we compared these results for stalks that act as linear springs with zero rest length to those that act as linear springs with non-zero rest length or as intrinsically non-linear freely jointed chains and worm-like, see Fig. 3. Within a force equilibrium model we find that for a linear motor stalk elasticity with a zero rest length within the gliding plane the step size distribution should be double- (or multiple) Gaussian distributions in agreement with our simulations. This provides an experimental test for a linear motor stalk elasticity with zero rest length. The experimental results of Ref. [1] show that indeed, double-Gaussian fits describe the experimental data well.

Furthermore, effects from non-linearity are important if forces 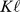 generated in single step drive the spring into a non-linear regime or if attachment energy of 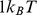 is sufficient to drive the spring into the non-linear regime. This is the case only if 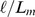 is sufficiently close to full stretching, which means larger than 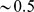 for freely jointed or worm-like chains, which is neither the case for truncated kinesin with 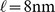 and 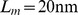 nor for myosin-V, which has a larger step length 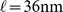 but also a larger contour length 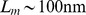. Therefore it is difficult to rule out such non-linear models based on the experimental data for step size distributions (Fig. 3).

On the other hand, our simulations show that a non-zero motor stalk rest length, which means a non-zero rest length for extensions within the gliding plane, has a much stronger effect and leads to a considerable broadening of the step size distributions for 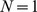 and 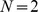. Moreover, for 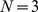 fractional steps become unobservable for kinesin-assays, i.e. the critical 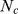 is reduced to 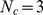 for a non-zero rest length as a result of the broadening of the step size distribution. The maximum of the step size distribution for 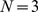 is around 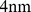 rather than 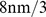 (Fig. 6). Therefore, reduction of experimental noise such that thermal noise is dominant, as in our simulations, would allow an experimental test for a non-zero motor stalk rest length by inspection of the step size distribution for 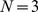.

For myosin-V gliding assays with the much larger step length 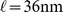 our simulations show that fractional 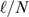 steps of F-actin are observable up to a much higher motor number 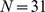 (Fig. 7). This agrees again very well with our criterion 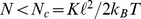 from eq. (9) for the observability of fractional filament steps in the presence of thermal noise, which gives 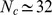 for the myosin-V gliding assay. This pronounced increase in the critical value 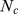 is caused by the quadratic dependence on the step length 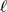. For an experimental measurement with additional noise we predict that fractional step up to 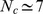 should be observable, which is still significantly higher as for kinesin assays. This prediction needs to be checked in further experiments. The critical value 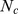 is also sensitive to the motor stalk stiffness 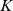. For a very small 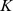 the critical number 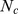 becomes small despite a large step size 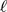. Our simulations confirm that 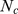 drops by a factor of 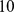 to 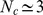 if the stiffness of the myosin-V stalk is reduced from 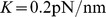 to 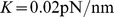. Therefore, an experiment on a myosin-V assay would also yield valuable information about the myosin-V stalk stiffness, which has been measured only in one experiment so far [12].

Using simulations for myosin-V gliding assays we also showed that fractional filament steps are still detectable in the filament step size distributions if the motor number 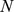 fluctuates around a certain average motor number 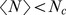, which is the typical experimental situation for gliding assays with a certain surface density (or coverage) of randomly adsorbed motors. The filament step size distributions are broadened but exhibit a pronounced peak at a step size slightly smaller than 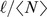.
